# Regulation of pro-apoptotic and anti-apoptotic factors in obesity-related esophageal adenocarcinoma

**DOI:** 10.1007/s11033-024-09931-6

**Published:** 2024-10-12

**Authors:** Swati Agrawal, Anna Podber, Megan Gillespie, Nick Dietz, Laura A. Hansen, Kalyana C. Nandipati

**Affiliations:** 1 School of Medicine, Creighton University, 2500 California Plaza, Omaha, NE 68178 USA; 2 Department of Surgery, School of Medicine, Creighton University, 7710 Mercy Road, Education Building, Suite 501, Omaha, NE 68124 USA; 3 Department of Pathology, School of Medicine, Creighton University, 7710 Mercy Road, Education Building, Suite 501, Omaha, NE 68124 USA; 4 Department of Biomedical Sciences, Creighton University, 2500 California Plaza, Omaha, NE 68178 USA

**Keywords:** Apoptosis, Inflammation, Obesity, Esophageal adenocarcinoma, Barretts esophagus

## Abstract

**Background:**

Obesity is a risk factor for esophageal adenocarcinoma (EAC). It was reported that obesity -associated inflammation correlates with insulin resistance and increased risk of EAC. The objective of the study is to investigate the role of obesity associated inflammatory mediators in the development of EAC.

**Methods:**

We included 23 obese and nonobese patients with EAC or with or without Barrett’s esophagus (BE) after IRB approval. We collected 23 normal, 10 BE, and 19 EAC tissue samples from endoscopy or esophagectomy. The samples were analyzed for the expression levels of pro-apoptotic and anti-apoptotic factors, PKC-δ, cIAP2, FLIP, IGF-1, Akt, NF-kB and Ki67 by immunofluorescence and RT-PCR. We compared the expression levels between normal, BE, and EAC tissue using Students’ *t-test* between two groups.

**Results:**

Our results showed decreased gene and protein expression of pro-apoptotic factors (bad, bak and bax) and increased expression of anti-apoptotic factors (bcl-2, Bcl-xL) in BE and EAC compared to normal tissues. There was increased gene and protein expression of PKC-δ, cIAP2, FLIP, NF-kB, IGF-1, Akt, and Ki67 in BE and EAC samples compared to normal esophagus. Further, an increased folds changes in mRNA expression of proapoptotic factors, antiapoptotic factors, PKC-δ, IGF-1, Akt, and Ki-67 was associated with obesity.

**Conclusion:**

Patients with EAC had increased expression of cIAP2 and FLIP, and PKC-δ which is associated with inhibition of apoptosis and possible progression of esophageal adenocarcinoma.

**Supplementary Information:**

The online version contains supplementary material available at 10.1007/s11033-024-09931-6.

## Introduction

Esophageal cancer ranks eighth in the global cancer incidence and considered to be the sixth most lethal cancer [1, 2]. The adenocarcinoma of the esophagus has been on the rise and became the most prevalent subtype in the West [3]. The rise in esophageal adenocarcinoma (EAC) is attributed largely to the obesity epidemic and reflux disease. Despite recent advances in treatment, the five-year survival rate for EAC remains low at 20.1% [4]. This growing incidence and persistently low survival rate underscore the need for more research into the molecular pathways of EAC to pave the way for better therapeutic options. Notably, obesity, a risk factor for EAC, has been associated with increased stress-related apoptosis and programmed cell death [4–6].

**Fig. 1:**
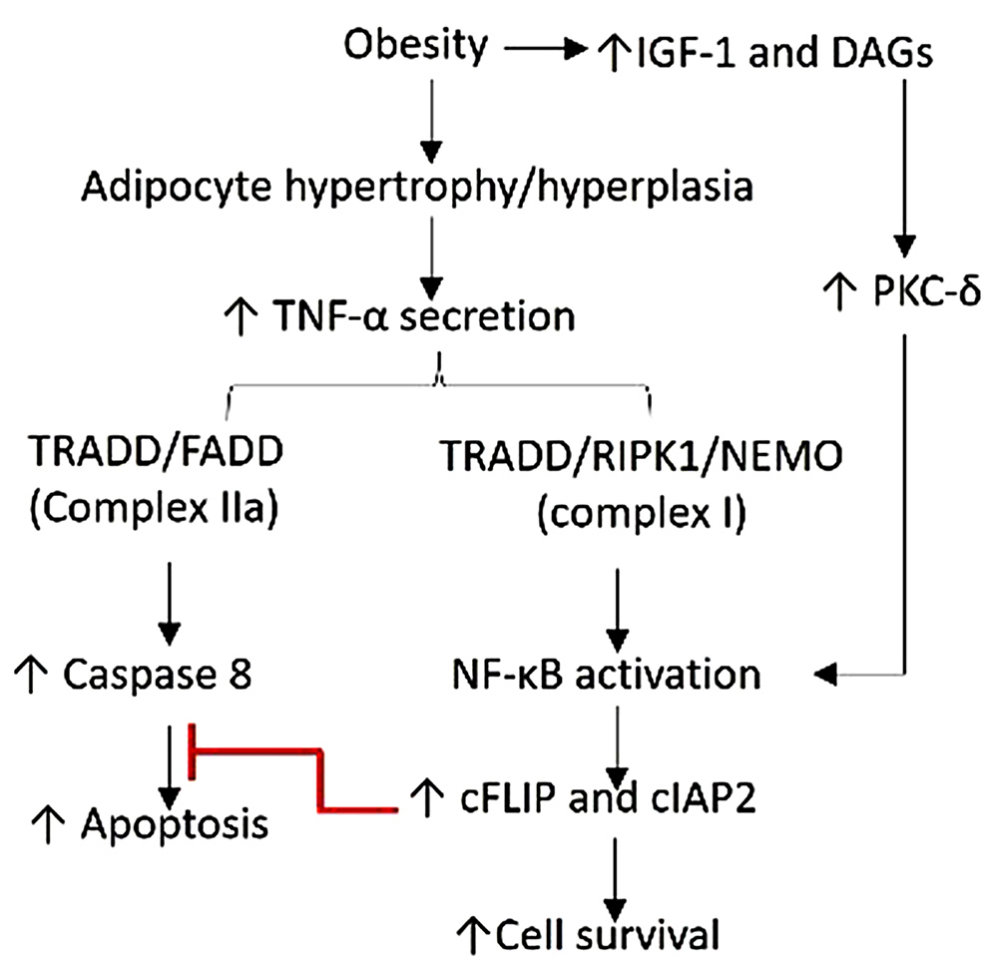
Schematic representation of the regulation of apoptosis involving cIAP2 and FLIP and their involvement in obesity associated esophageal adenocarcinoma

Obesity -associated inflammation mediated by an increased expression of triggering receptor expressed on myeloid cells (TREM-1), high mobility group box protein (HMGB)-1, toll- like receptor (TLRs), receptor for advanced glycation end products(RAGE), and increased infiltration of immune cells correlates with insulin resistance [7–9]. Obesity-associated free fatty acids (FFA) changes can lead to increased expression of insulin growth factor-1 (IGF-1) and diacylglycerol (DAG). DAG enhances the expression of protein kinase C δ (PKC-δ), a serine-threonine kinase that acts as a pleiotropic regulator of cell proliferation, differentiation, and survival [10–12]. PKC-δ is also involved in cytokine-mediated inflammation in obesity [13]PKC-δ is also involved in cytokine -mediated inflammation in obesity [13]. This suggests that the association of PKC-δ and inflammatory mediators in obesity may be associated with increased risk of EAC because EAC has an inflammatory etiology [14]Further, activation of various signaling pathways regulates cell apoptosis [15] an important cellular event in carcinogenesis. EAC cells can circumvent apoptosis through an obesity-induced IGF1-DAG-PKC-δ pathway, or independently of the PKC-δ pathway via modifications to downstream regulators. One proposed mechanism of such downstream regulation is believed to be mediated by the cellular inhibitor of apoptosis 2 (cIAP2) and cellular FLICE-inhibitory proteins (c-FLIP).

c-FLIPs are considered mediators of anti-apoptotic pathways, inhibiting programmed cell death. Similarly, human IAPs, including cIAP1, cIAP2, and XIAP, regulate apoptosis by negatively regulating ripoptosomes and activating apoptotic and necroptotic cell death responses [16, 17]. In esophageal squamous cell cancers, cIAP2 expression is higher in cancerous tissue compared to normal mucosa [18], suggesting a potential association with cancer progression [19]. However, the interaction between PKC-δ and downstream signaling involving apoptotic inhibitory proteins (cIAP2 and c-FLIP), which may play a pivotal role in malignant cell proliferation, has not been thoroughly investigated in EAC (Fig. 1). The aim of our study is to discern the relationship between the expression of PKC-δ, inflammatory mediators and downstream cIAP2 and c-FLIP, and to determine the impact of this relationship on apoptosis in EAC.

## Materials and methods

### Patient selection

This prospective study received approval from the Institutional Review Board (IRB) of Creighton University (IRB No. 1194896). Based on the power analysis using G*power3.0.10 software with an α value of 0.05, the sample size necessary to have at least 95% power to detect significant change is 10 in each group. Following IRB approval, informed consent was obtained, and 23 patients were recruited from the surgery clinics at Creighton University Medical Center and CHI Health Immanuel Medical Center. The inclusion criteria encompassed patients aged 19 or older with a clinical diagnosis of esophageal cancer, which was confirmed through endoscopic biopsy. The exclusion criteria ruled out patients aged 18 or younger, those unwilling to participate in the study, and biopsies that did not confirm EAC. During the study, 23 normal tissue samples (from regular esophageal lining), 10 Barrett’s Esophagus samples, and 19 EAC samples were collected from 23 recruited patients. Patient weight was recorded either at the time of the endoscopy or surgery. Obesity was defined as a BMI greater than 30 kg/m^2^. Demographics and clinical data were sourced from patient charts. We collected variables such as age, sex, body mass index (BMI), EAC staging, medication usage, smoking and alcohol consumption history, and co-morbid conditions. A power analysis determined the sample size among groups based on BMI, achieving a power (1-β) of 95% and α set at 0.05. The minimum number of samples required in each group to achieve statistical significance between groups was identified as 10.

### Tissue collection and processing

Esophageal tissue biopsies, both from tumor and non-tumor regions, were obtained during endoscopy or surgery. These samples were promptly transported to the Creighton University lab in either formalin, University of Wisconsin solution, or RNA later solution, and they were kept at 4 °C. Tissue samples preserved in RNA later were stored at -80 °C for RNA isolation. A board-certified pathologist assessed each tissue sample to determine the presence or absence of EAC. For histological analysis, a segment of each esophageal tissue sample was preserved in 10% buffered formalin for 24 h. Subsequently, the tissue samples underwent processing with the Excelsior ES tissue processor (Thermo Scientific, USA) through various cycles of dehydration in ethanol baths, followed by paraffin baths. The tissues were then embedded in paraffin blocks. Thin tissue sections, measuring 5 μm, were cut using a Leica RM 2135 microtome and then mounted on glass slides. These slides were placed in a 72 °C oven to melt the paraffin wax, a process that lasted for 20 min.

### Hematoxylin and eosin stain

Hematoxylin and Eosin (H&E): staining was performed following the standard protocol in our laboratory. Briefly, the tissue sections on the slides were de-paraffinized, rehydrated in ethanol, rinsed in double-distilled water, and stained with hematoxylin for 45 s and with eosin for 30 s. The stained sections were mounted with a xylene-based mounting medium and a coverslip was placed over the tissue. The stained tissue sections were examined under a light microscope (Leica DM6) and the images were scanned with a scale of 100 μm. We stained at least three adjacent sections from each tissue and three images were scanned from each section for analysis.

### Immunofluorescence assays

At the Creighton University laboratory, samples were prepared as per standard protocol for immunofluorescence assays via deparaffinization and rehydration. Antigen retrieval was performed by heating the section in DAKO Target Retrieval solution for 1 h. Briefly, after antigen retrieval, the slides were cooled down to room temperature and washed with 1X phosphate buffered saline (PBS) three times for 5 min each. This was followed by blocking the nonspecific antigens with blocking buffer for one hour at room temperature followed by the incubation with primary antibodies, rabbit anti-Bad (ab45782), rabbit Anti-Bak (ab32371), rabbit Anti- Bax (ab32503), rabbit Anti-Bcl-2 (ab32124), rabbit Anti- Bcl- XL (ab32370), rabbit Anti-PKC-𝛿 (ab182126), rabbit Anti- pan-Akt (ab8805), rabbit Anti-cIAP2 (ab23423), rabbit Anti-IGF1 (ab182408), rabbit Anti-FLIP (ab 8421), and rabbit Anti- NF-κB p65 (ab131109) and rabbit Anti-Ki67 (ab16667) overnight at 4^0^C in a dilution of 1:100. This was followed by PBS wash 3 times 5 min each and incubation with donkey anti rabbit Alexa Fluor 488 (green) Invitrogen A32790 Thermo Fisher Scientific conjugated secondary antibodies at 1:500 dilutions for 1 h at room temperature. The sections were washed with PBS while gently shaking. Nuclear staining was done with 4′,6-diamidino-2-phenylindole (DAPI). The slides were mounted with Antifade Gold reagent containing DAPI (H-1200; Vectashield, Vector labs). The slides were scanned with Nikon inverted fluorescent microscope at 100 μm. A minimum of three scanned images from each sample was used to estimate the fluorescence intensity using ImageJ (NIH) software and mean fluorescence intensity (MFI) was analyzed for each protein of interest. The fluorescence intensity measurement and MFI calculations were cross checked by two blinded reviewers.

### RNA isolation, cDNA preparation, and real-time PCR

Total RNA was isolated using TRI reagent (T9424, Sigma, St Louis, MO, USA). The yield of total RNA was measured using NanoDrop One (Thermo Fisher Scientific, USA). Further, the cDNA was synthesized using iScript cDNA synthesis kit (1708891 BioRad) and Real-Time PCR (RT-PCR) was performed in triplicate using SYBR Green Master Mix (#1708880, BioRad) using Real Time cycler (Applied Biosystems 7500 Fast Dx Real-Time PCR). The cycling conditions were 5 min at 95 °C for initial denaturation, 40 cycles of 30 s at 95 °C, 30s at 55–60 °C (based on primer annealing temperatures), and 30 s at 72 °C followed by melting curve analysis. The primers for *Bad*,* Bak*,* Bax*,* Bcl-2*,* Bcl-XL*, PKC-δ ,* Akt*,* cIAP2*,* IGF1*,* FLIP*, and *NF-κB* were obtained from Integrated DNA Technologies (Coralville, Iowa 52241.USA) and the forward and reverse nucleotide sequences are given in Table 1.

**Table 1:** Forward and reverse nucleotide sequence of the primers used in RT-PCR

Gene	Direction	Sequence
*Bax*	Forward	5′-CCCGAGAGGTCTTTTTCCGAG-3′
	Reverse	5′-CCAGCCCATGATGGTTCTGAT-3′
*Bak*	Forward	5′-TGCTAGTGCCCTCTCTCTGG-3′
	Reverse	5′-GTGGGAATGGGCTCTCACAA-3′
*Bcl-2*	Forward	5′-TCGCCCTGTGGATGACTGA-3′
	Reverse	5′-CAGAGACAGCCAGGAGAAATCA-3′
*Bcl-xL*	Forward	5′-TAAGGCGGATTTGAATCTC-3′
	Reverse	5′-ATAATAGGGATGGGCTCAAC-3′
*Bad*	Forward	5’-TAAGAAGGGACTTCCTCGCC-3’
	Reverse	5’-GTTCCGATCCCACCAGGACT-3’
*PKC-*δ	Forward	5′-GCATCTCCACGGAACGAC-3′
	Reverse	5′-CCACCTCCACCTTCTCAACT-3′
*AkT1*	Forward	5′-GGAGGTTTTTGGGCTTGCG-3′
	Reverse	5′-CTCTGATGCACCAGCTGACA-3′
*cIAP2*	Forward	5′-GCTTTTGCTGTGATGGTGGACTC-3′
	Reverse	5′-CTTGACGGATGAACTCCTGTCC-3′
*IGF1*	Forward	5′ACACAATCTGCCTCCCTCATTT3′
	Reverse	5′AGTCCCTTCAGGGGCTTTCA3′
*FLIP*	Forward	5′AGTGAGGCGATTTGACCTGCTC3′
	Reverse	5′ CCTCACCAATCTCTGCCATCAG3’
*NF-κBp65*	Forward	5’-GACTACGACCTGAATGCTGTG-3’
	Reverse	5’-GTCAAAGATGGGATGAGGAAGG-3’
*Ki67*	Forward	5’-CTTTGG GTG CGA CTT GAC G-3’
	Reverse	5’-GTCGACCCCGCTCCTTTT-3’
*β-actin*	Forward	5’-CCTGGCACCCAGCACAAT-3’
	Reverse	5’-GCCGATCCACACGGAGTACT-3’

Correlation analysis: We analyzed the data of gene expression of various genes of interest (standardized to housekeeping gene; CT gene- CT beta actin) for correlation between non-obese and obese patients using Excel Data Analysis tool.

### Statistical analysis

Data is presented as the mean ± SD. Data was analyzed using GraphPad Prism 9. The comparison between two groups for the expression of the protein of interest was performed using One-way ANOVA with Bonferroni’s post-hoc correction. A probability (*p*) value of < 0.05 was accepted as statistically significant. *p < 0.05, **p < 0.01, ***p < 0.001 and ****p < 0.0001.

## Results

**Table 2:** Patient demographics are listed in table and noted to have male predominance

Patient Demographics (*n* = 23)	Mean (range)
Age	63 years (48 year − 80 year)
Sex	19 males 4 females
BMI - mean (range)	27.83 kg/m^2^ (19.76–40.5)
EAC staging Stage I Stage II% Stage III Stage IV	4% 13% 52 14%
Current or Prior BE (%)	43.5%
Current Proton Pump Inhibitor use (%)	87%
Tobacco use (%)	91.3%
Alcohol use (%)	56.5%

Hematoxylin and eosin (H&E) staining revealed increased inflammation, fibrosis, moderately differentiated lesions in EAC. (Supplementary Fig. 1)

### The fold change in mRNA expression of pro-apoptotic factors were decreased while of anti-apoptotic factors were increased in esophageal adenocarcinoma compared to normal esophagus

**Fig. 2:**
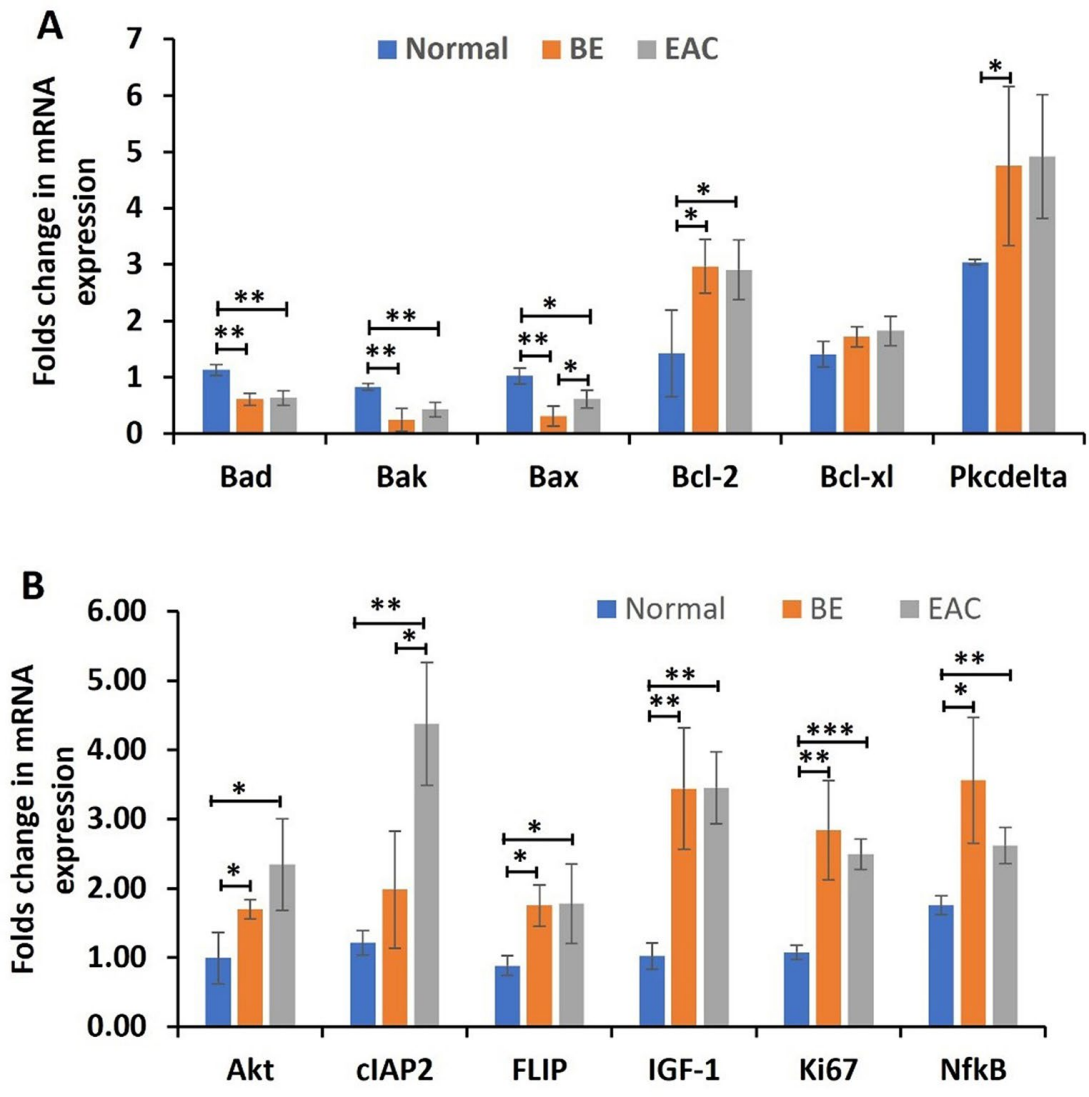
RT-PCR for proapoptotic (Bad, Bak, Bax), antiapoptotic (Bcl-2, Bcl-XL), protein kinase C delta (PKC-δ), cellular inhibitor of apoptosis 2 (cIAP2), FLICE-like inhibitory protein (FLIP), protein kinase B (Akt), insulin like growth factor 1 (IGF-1), proliferation marker Ki67, and nuclear factor kappa beta (NF-κB) in normal, BE, and EAC tissues. Data are presented as the mean ± SD (*n* = 3; biological replicates). *p < 0.05, **p < 0.01, ***p < 0.001 and ****p < 0.0001. The data represents a total of 23 normal samples, 10 Barrett’s Esophagus samples, and 19 EAC samples

The RT-qPCR results showed a decreased fold change in mRNA expression of pro-apoptotic markers Bad, Bak and Bax in Barrett’s Esophagus and EAC as compared to the normal esophagus. The fold changes in mRNA expression of Bad (*p* = 0.003 and *p* = 0.009) and Bak (*p* = 0.005 and *p* = 0.008) were significantly decreased while was increased for Bax (*p* = 0.005and *p* = 0.03) in BE and in EAC tissues compared to the normal esophageal tissues. There were no significant differences between the fold changes in mRNA expression of Bad, Bak and Bax between BE and EAC (Fig. 2 panel A).

The RT-qPCR results showed The fold change in mRNA expression of anti-apoptotic markers Bcl2 and Bcl-xL was significantly increased in BE (*p* = 0.04 and *p* = 0.13) and EAC tissues (*p* = 0.05 and *p* = 0.10) compared to the normal esophageal tissues while there were no significant differences for Bcl2 and BcL-xL between BE and EAC (Fig. 2 panel A). Overall, the results showed decreased expression of apoptotic factors and increased expression of anti-apoptosis factors, which impair apoptosis and may contribute to BE and EAC carcinogenesis.

### RT-PCR showed increased mRNA expression of PKC-δ, IGF-1, Akt, NF-κB, cIAP2, FLIP, and Ki67 in BE and EAC compared to normal tissue samples

The qRT-PCR analysis revealed significantly increased folds change in mRNA expression of PKC-δ, IGF-1 (*p* = 0.009 and *p* = 0.001), Akt (*p* = 0.03 and *p* = 0.03), NF-κB (*p* = 0.02 and *p* = 0.007), and proliferation marker Ki67 (*p* = 0.01 and *p* = 0.0004) in BE and EAC compared to normal esophageal tissue (Fig. 2 panel B). The folds change in mRNA expression of PKC-δ was significantly increased in EAC compared to normal (*p* = 0.04; 4.91 vs. 3.04) and increased in BE (*p* = 0.11; 4.76 vs. 3.04). However, there was no significant difference between the BE and EAC (Fig. 2 panel B).

The folds change in mRNA expression of cIAP2 was significantly increased in EAC (*p* = 0.003) but was not significant in BE (*p* = 0.19) compared to the normal esophageal tissues while was significantly increased between BE and EAC (*p* = 0.02). The fold change in mRNA expression for FLIP was significantly increased in BE and (*p* = 0.01) EAC (*p* = 0.05) when compared to the normal tissues samples while there was no significance difference between BE and EAC (Fig. 2 panel B).

### Immunofluorescence showed decreased immunopositivity for pro-apoptotic mediators Bad, Bak, and Bax while there was increased immunopositivity for anti-apoptotic factors Bcl-2 and Bcl-xL and PKC-δ in EAC

**Fig. 3:**
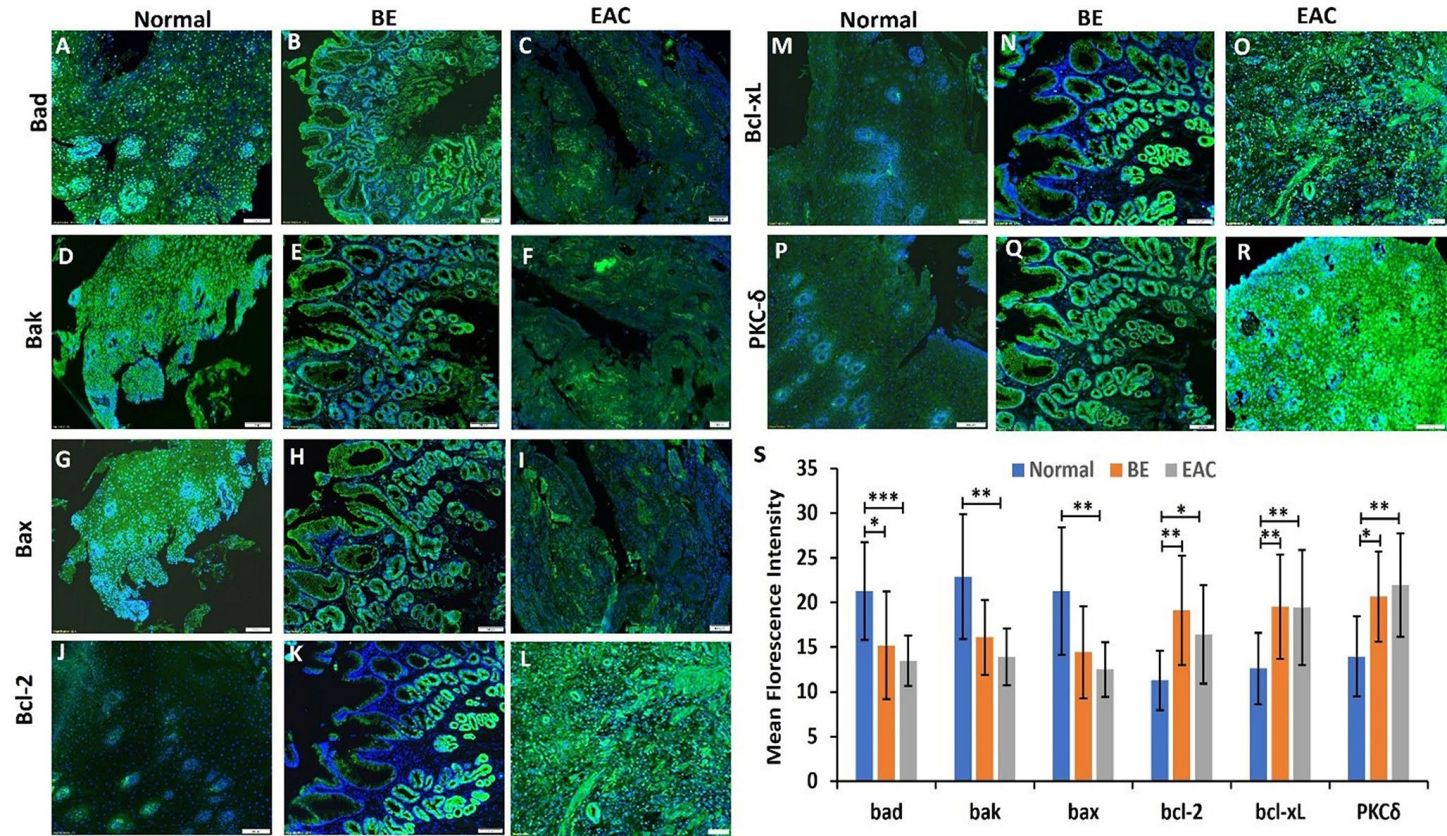
Immunofluorescence staining for Bad, Bak, Bax, Bcl-2, Bcl-xL and PKC-δ in normal, BE, and EAC tissues. Data are presented as the mean ± SD (*n* = 3 biological replicates). *p < 0.05, **p < 0.01, ***p < 0.001 and ****p < 0.0001. These are representable images from a total of 23 normal samples, 10 Barrett’s Esophagus samples, and 19 EAC samples

Immunofluorescence showed decreased immunopositivity for proapoptotic factors Bad, Bak and Bax in EAC and BE tissues compared to normal. (Fig. 3 panels A, B, C, D, E, F, G, H, and I). The mean fluorescent intensity (MFI) for Bad was significantly decreased in BE and EAC compared to normal esophageal tissue (Fig. 3 panel S) while MFI for Bak and Bax was significantly decreased in EAC compared to normal (Fig. 3 panel S). These results suggests that proapoptotic factors are downregulated during EAC tumorigenesis. The immunopositivity of anti-apoptotic factors Bcl-2 and Bcl-xL was increased in EAC as compared to BE and normal esophagus (Fig. 3 panels J, K, L, M, N, and O). The IF of PKC-δ showed increased immunopositivity in EAC as compared to BE and normal (Fig. 3 panels P, Q, and R). The MFI of Bcl-2, Bcl-xL, and PKC-δ was significantly higher in BE and EAC compared to normal tissues (Fig. 3 panel S).

### Immunofluorescence showed increased immunopositivity for cIAP2, FLIP, IGF-1, Akt, NF-κB, and Ki67 in esophageal adenocarcinoma

**Fig. 4:**
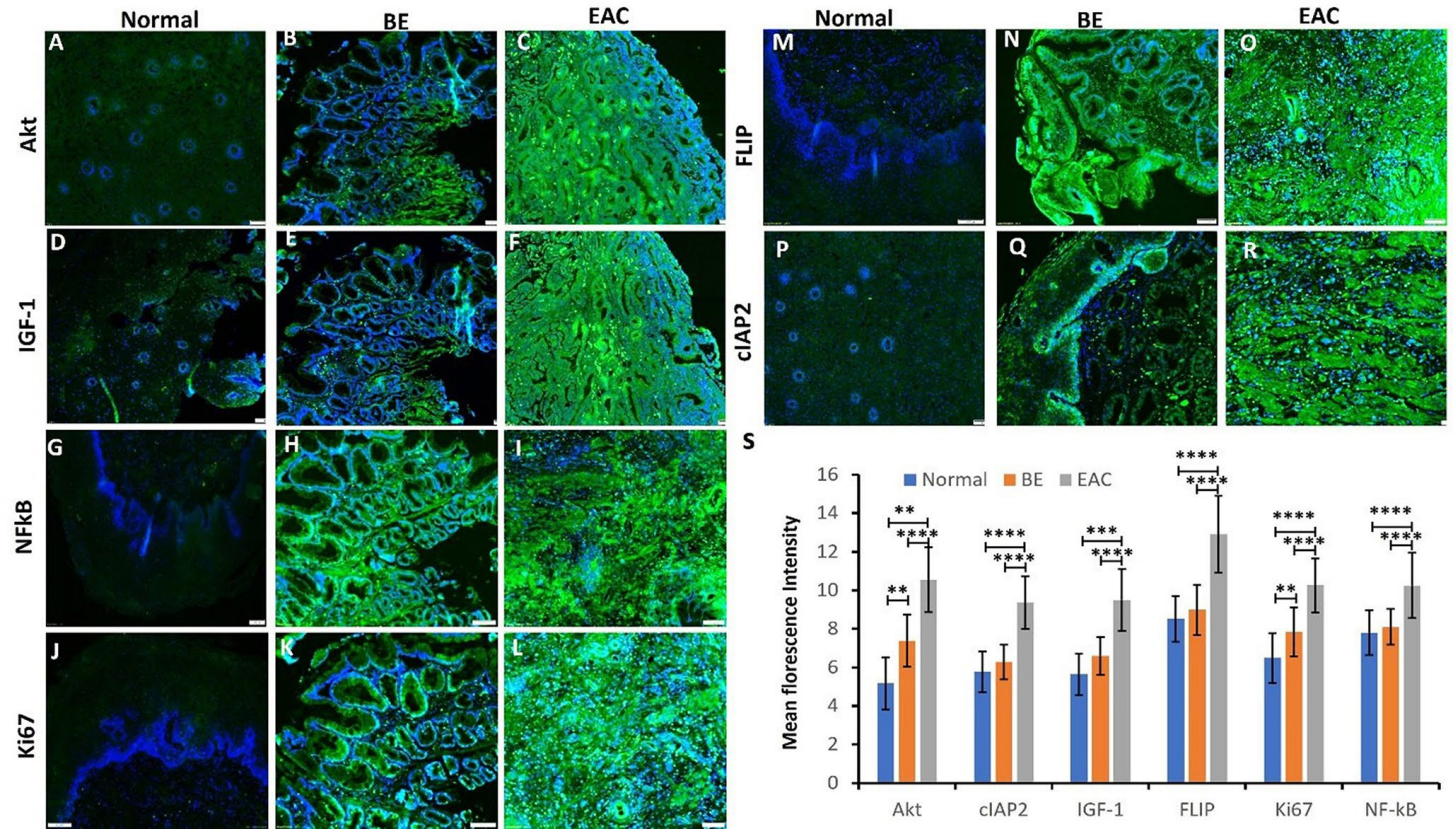
Immunofluorescence for cIAP2, FLIP, IGF-1, Akt, NF-κB, and Ki67 in normal, BE, and EAC tissues. Data are presented as the mean ± SD (*n* = 3 biological replicates). *p < 0.05, **p < 0.01, ***p < 0.001 and ****p < 0.0001. These are representable images from a total of 23 normal samples, 10 Barrett’s Esophagus samples, and 19 EAC samples

Immunopositivity of Akt, IGF-1, NF-κB, and Ki-67 was increased in EAC compared to BE and normal esophageal tissue (Fig. 4 panels A, B, C, D, E, F, G, H, I, J, K and L). The MFI of Akt was significantly increased in BE and EAC compared to normal tissue and in EAC compared to BE (Fig. 4 panel S). The MFI for IGF-1 and NF-κB was significantly increased in BE and EAC compared to normal tissue (Fig. 4 panel S) while the MFI for Ki-67 was significantly increased in BE and EAC compared to normal tissue and in EAC compared to BE (Fig. 4 panel S). The immunopositivity (Fig. 4 panel M, N, O, P, Q, and R) and MFI (Fig. 4 panel S) for cIAP2 and FLIP was increased in BE and EAC as compared to normal esophagus. These results indicate the association of increased expression of PKC-δ, FLIP, and cIAP2 in EAC as compared to normal and BE.

### Obesity is associated with an increased folds changes in mRNA expression of proapoptotic factors, antiapoptotic factors, PKC-δ, IGF-1, Akt, and Ki-67 and decreased folds change in cIAP2 and FLIP in normal tissues

**Fig. 5:**
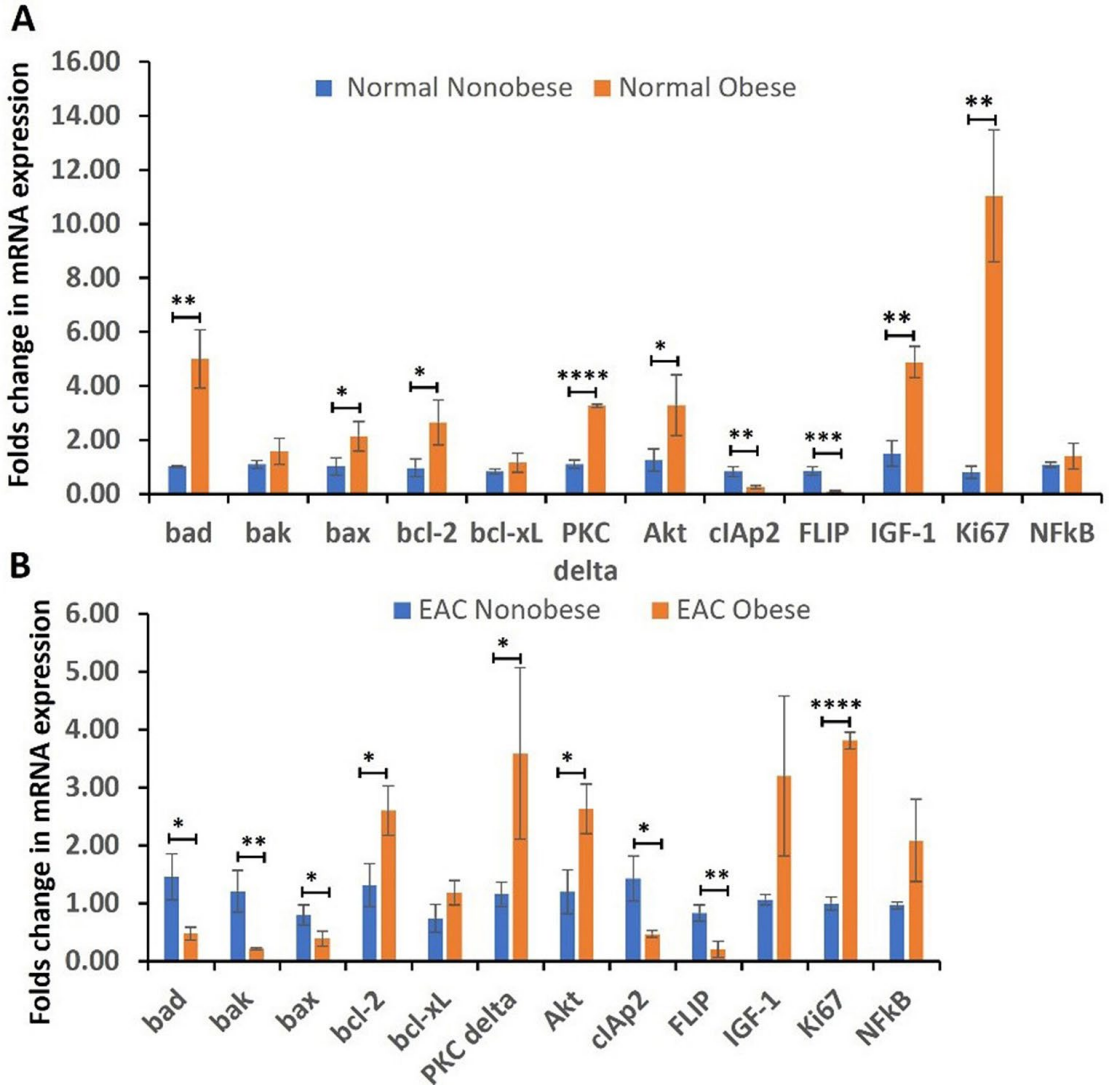
RT-PCR for proapoptotic (Bad, Bak, Bax), antiapoptotic (Bcl-2, Bcl-XL), protein kinase C delta (PKC-δ), cellular inhibitor of apoptosis 2 (cIAP2), FLICE-like inhibitory protein (FLIP), protein kinase B (Akt), insulin like growth factor 1 (IGF-1), proliferation marker Ki67, and nuclear factor kappa beta (NF-κB) in normal and EAC tissues of obese and non-obese subjects. Data are presented as the mean ± SD (*n* = 3 biological replicates). *p < 0.05, **p < 0.01, ***p < 0.001 and ****p < 0.0001. The data represents a total of 23 normal samples, 10 Barrett’s Esophagus samples, and 19 EAC samples

RT-PCR analysis revealed that the fold change in mRNA expression of Bad, Bax, and Bcl-2 (*p* = 0.003, *p* = 0.039, and *p* = 0.032, respectively) was significantly elevated in normal esophageal tissue samples of the obese group compared to nonobese group. However, it was not significantly increased in Bak and Bcl-xL (*p* = 0.16 and *p* = 0.19) (Fig. 5 panel A). The folds change in mRNA expression of IGF-1, Akt, and Ki-67 was significantly increased (*p* = 0.001, *p* = 0.04, and *p* = 0.001, respectively) while the expression of NF-kB was not significantly increased (*p* = 0.31) in normal tissue samples of obese compared to non-obese subjects. Surprisingly, the folds change in mRNA expression of cIAP2 and FLIP was significantly decreased (*p* = 0.008 and *p* = 0.0008) in obese compared to non-obese normal samples (Fig. 5 panel A).

### Obesity is associated with decreased expression of proapoptotic genes, cIAP2, and FLIP while increased expression of antiapoptotic genes, PKC-δ, Akt, IGF-1, Ki-67, and NF-κB in EAC tissues

The folds change in mRNA expression of proapoptotic genes Bad, Bak and Bax was significantly decreased (*p* = 0.01, *p* = 0.008 and *p* = 0.03 respectively), for Bcl-2 was significantly increased (*p* = 0.01), and increased folds change of Bcl-XL was not statistically significant (*p* = 0.07) in obese patients with EAC compared to non-obese EAC tissues (Fig. 5 panel B). The fold change in mRNA expression of PKC-δ, Akt, IGF-1, and NF-kB was significantly increased (*p* = 0.04, *p* = 0.01, *p* = 0.05, and *p* = 0.05 respectively) in obese EAC patients compared to non-obese EAC tissues. Surprisingly, the fold change in mRNA expression of cIAP2 and FLIP were significantly decreased (*p* = 0.01 and *p* = 0.005 respectively) in obese EAC patients as compared non-obese EAC tissues (Fig. 5 panel B). These findings suggest the association of obesity with EAC tumorigenesis.

### Correlation analysis reveals a positive association of various factors with obesity

**Fig. 6:**
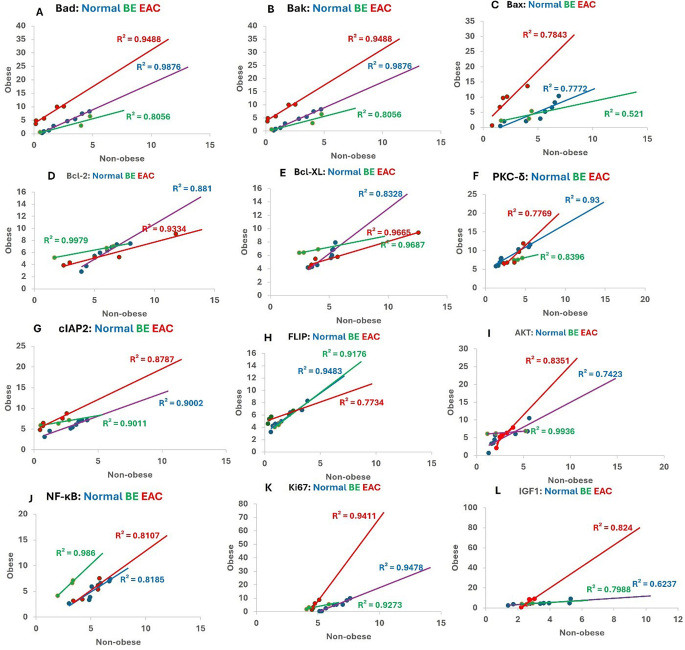
Correlation analysis of various factors with obesity. Protein kinase C delta (PKC-δ), cellular inhibitor of apoptosis 2 (cIAP2), FLICE-like inhibitory protein (FLIP), protein kinase B (Akt), insulin like growth factor 1 (IGF-1), proliferation marker Ki67, and nuclear factor kappa beta (NF-κB) in normal and EAC tissues of obese and non-obese subjects

The correlation analysis revealed a positive correlation of apoptotic factors Bad, Bak, and Bax (Fig. 6 panels A, B, and C), anti-apoptotic factors Bcl-2 and Bcl-XL (Fig. 6 panels D and E), PKC-δ (Fig. 6 panel F), inflammatory mediators AKT and NF-κB (Fig. 6 panels I and J), IGF-1 (Fig. 6 panel L), proliferation marker Ki-67 ( Fig. 6 panel K), and regulators of apoptosis cIAP2 and FLIP (Fig. 6 panels G and H) with obesity. The results revealed a strong positive correlation for all genes with obesity in EAC except for Bcl-2 and Bcl-XL where there was a strong correlation but mor towards the non-obese patients.

## Discussion

Our study found increased expression of anti-apoptotic genes (Bcl-2 and Bcl-xL) and upstream apoptosis regulators (cIAP2 and FLIP), along with decreased expression of pro-apoptotic genes (Bad, Bax, and Bak), in both Barrett’s esophagus (BE) and esophageal adenocarcinoma (EAC) tissues compared to normal esophageal tissues from obese patients. Apoptotic signaling plays a vital role in maintaining a balance between cell death and survival, as well as preserving genome integrity. Dysregulation of apoptotic and anti-apoptotic factors stands out as a prominent characteristic of cancer [20]. The overexpression of anti-apoptotic BCL-2 family proteins is frequently observed in cancer cells [21]. Moreover, the overexpression of anti-apoptotic proteins has been associated with cancer recurrence, poor prognosis, and resistance to cancer therapeutics [22]. Our results of increased levels of pro-apoptotic factors and dysregulated apoptosis, particularly in EAC tissues compared to normal esophageal tissues suggest that dysregulated apoptosis could be a significant underlying mechanism in the development and progression of EAC. However, the specific regulation of pro-apoptotic and anti-apoptotic factors, particularly in the context of obesity induced EAC development, remains elusive.

Obesity has emerged as a significant risk factor for various cancers, including endometrial cancer, colorectal cancer, postmenopausal breast cancer, prostate cancer, renal cancer, and esophageal adenocarcinoma, with approximately 20% of all cancer cases attributed to excess weight [23–26]. Obesity also reported to contribute to tumor growth and progression due to increased levels of free fatty acids and dietary lipids [27, 28]. The overexpression of anti-apoptotic factors and the inhibition of proapoptotic factors, in cases of BE and EAC among obese patients compared to non-obese individuals indicates the importance of obesity-associated elevated levels of free fatty acids and dietary lipids, in the development of BE and EAC [29–32]. The correlation of obesity with increased incidences of EAC is supported by a positive correlation of PKC-δ and IGF-1 with obesity in BE and EAC. Furthermore, with obesity, the inability of adipose tissues to adequately expand triggers adipocyte apoptosis and the development of insulin resistance [33]. Anti-apoptotic therapy is useful in such conditions, and a decrease in the correlation of Bcl-2 and Bcl-XL with obesity suggests decreased anti-apoptosis with obesity. Since the development of EAC is associated with the proliferation of tumor cells, a positive correlation of Ki-67 with obesity in BE and EAC supports our hypothesis that obesity contributes to the EAC cascade. Additionally, a positive correlation of cIAP2 and FLIP with non-obese individuals compared to those with obesity supports our hypothesis that a decrease in regulators of apoptosis contributes to EAC pathogenesis.

The essential mechanism underlying the obesity associated malignancy remain elusive; however, alterations in the IGF-axis have been proposed as a potential mechanism for carcinogenesis [34–38]. Our study revealed increased levels of IGF-1 in BE and EAC obese tissues, increased expression of Ki-67 (a marker of cell proliferation) in BE and EAC tissues, and decreased proliferation and migration of EAC cells with IGF1 inhibition. These findings suggest a robust relationship between obesity and EAC carcinogenesis and are consistent with the notion that insulin, insulin resistance, and insulin-like growth factor (IGF)-1 play significant roles in cell proliferation, differentiation, and apoptosis, contributing to carcinogenesis [39]. This makes them intriguing targets for cellular studies linking obesity and cancer [40] as well as for therapeutics. However, the precise molecular mechanisms and downstream signaling pathways involved in this relationship warrant further investigations.

Elevated levels of IGF-1 in obesity and the subsequent dysregulation of downstream signaling pathways have been implicated in cancer progression, particularly in squamous cell carcinoma of the esophagus [40]. Further, downstream cell cycle regulators such as c-FLIP and cIAP2 regulating apoptosis mediated by TRAIL and CD95L in various cancers [41] makes c-FLIP and cIAP2 as promising target for cancer therapy. Combining c-FLIP inhibition with other treatments, such as TRAIL or conventional chemotherapy, could enhance its effectiveness [42, 43]. c-FLIP has been identified as a key negative regulator of apoptosis in human cancer cells, and its expression is controlled by several transcription factors, including AP-1 (c-Fos and c-Jun), CREB, SP1, and NF-kB. In our study, we observed increased expression of c-FLIP and cIAP2 in both BE and EAC tissues compared to normal esophageal tissues, which was accompanied by decreased expression of pro-apoptotic markers Bax, Bak, and Bad (Figs. 2 and 3, and 4). These findings suggest a negative association between c-FLIP and cIAP2 and pro-apoptotic gene regulation. One possible underlying mechanism is the inhibition of caspase-8 mediated apoptosis [44] (Fig. 1). It is worth noting that the role of c-FLIP and cIAP2 in EAC patients have not been extensively studied in the literature and investigating the regulatory role of cFLIP and cIAP2 in apoptosis in the context of EAC may be helpful in designing better therapeutics for EAC [41–45] .

Protein kinase C (PKC), a family of phospholipid-dependent serine/threonine protein kinases, regulates a wide variety of cellular functions, including cell proliferation, differentiation, and cell death [46]. Our results showed overexpressed PKC-δ in BE and EAC compared to the normal tissues in obese patients compared to nonobese patients. This suggests that increased PKC-δ which regulates cFLIP and cIAP2 expression through NF-κB, is associated with EAC progression in obesity. Our results are unique since increased PKC-δ expression and its correlation with cFLIP and cIAP2 expression in obesity has not been reported widely in the literature.

The secretion of inflammatory cytokines (IL-1, IL-6, and TNF-α) from infiltrated immune cells, mainly macrophages, is associated with obesity [47–49] and may exert control over apoptotic mediators through upstream regulation of cIAP2 and FLIP involving NF-κB, a transcription factor that is activated by cytokines secreted in obesity (Fig. 1). An increased expression of NF-κB in both BE and EAC tissues, along with cFLIP and cIAP2 suggest that the upregulation of inflammatory cytokines, TNF-α primarily, activates cFLIP and cIAP2. Moreover, TNF-α triggers the activation of caspase-8 and NF-κB [50, 51], which subsequently activate apoptosis, cFLIP, and cIAP2 through independent pathways (Fig. 1). TNF-α simultaneously stimulates pro-apoptotic and anti-apoptotic signals, and cellular death occurs when the anti-apoptotic signals, mainly mediated by NF-κB activation, are suppressed [51] (Fig. 1). However, further in vitro studies are necessary, involving the blocking of complex I [52] and complex II and stimulation with TNF-α, to provide additional support for the molecular mechanisms involved for EAC tumorigenesis.

Additionally, besides NF-κB, the stimulation of growth factors or activation of other pathways, such as mitogen-activated protein kinase (MAPK) and the phosphatidylinositol-3 kinase (PI3K)/Akt, can induce the expression of c-FLIP and hinder apoptosis triggered by death receptors [53]. Notably, our findings of upregulation of cytoplasmic kinase Akt in the tissues of BE and EAC compared to normal esophageal tissues from obese patients strengthen the hypothesis that inflammatory signaling activation in obesity regulates apoptosis through the activation of cIAP2 and FLIP. Consequently, targeting cIAP2 and FLIP holds potential as a therapeutic approach for EAC. It is important to acknowledge the limitations of our study, including a small sample size and a lack of in-vitro studies to establish a direct causal relationship between PKC-δ and apoptotic pathways. Nevertheless, our results demonstrate significantly elevated levels of PKC-δ, cIAP2, and FLIP in BE and EAC patients compared to normal esophageal tissues from obese patients pave the way to investigate the role of cIAP2, and FLIP in EAC and therapeutic potential of targeting these molecules. The findings of this study contribute to a better understanding of the molecular pathways involved in obesity-induced esophageal cancer and may offer insights into potential therapeutic targets. It should be noted that RT-PCR and immunofluorescence measures the expression of gene and protein, respectively, but not the activity of the proteins analyzed in this study.

## Conclusion

Significantly increased expression of PKC-δ and inflammatory mediators with a significant association between the expression of apoptotic regulators cIAP2 and FLIP and pro- and anti-apoptotic markers in BE and EAC tissues suggest that dysregulated programmed cell death, in part, is associated with EAC carcinogenesis and targeting cIAP2 and FLIP seems to be a promising strategy to attenuate the progression of BE and EAC. This study investigated the correlation between inflammation associated dysregulated apoptosis in obesity with EAC progression, one piece of the puzzle, with other etiologies including endocrine, mechanical and microbiological factors.

## Electronic supplementary material

Below is the link to the electronic supplementary material.

Supplementary Material 1

## Data Availability

No datasets were generated or analysed during the current study.

## Abbreviations

EAC: Esophageal Adenocarcinoma
BE: Barrett’s Esophagus
Akt: Protein Kinase B
cIAP2: Cellular Inhibitor of Apoptosis 2
PKC δ: Protein Kinase C delta
TNF-α: Tumor Necrosis Factor alpha
IL: Interleukin
cFLIP: CASP8 and FADD-like apoptosis regulator
(IGF)-1: Insulin like growth factor
NF-κB: Nuclear factor kappa beta
MAPK: Mitogen-activated protein kinase
PI3K: phosphatidylinositol-3 kinase
