# Supplementary material for: Regulation of pro-apoptotic and anti-apoptotic factors in obesity-related esophageal adenocarcinoma
Source: Mol Biol Rep. 2024 Oct 12;51(1):1049. doi: 10.1007/s11033-024-09931-6 (PMC11470870; doi:10.1007/s11033-024-09931-6)
Supplement: Supplementary file 1 — Supplementary Material 1 [file 11033_2024_9931_MOESM1_ESM.docx]

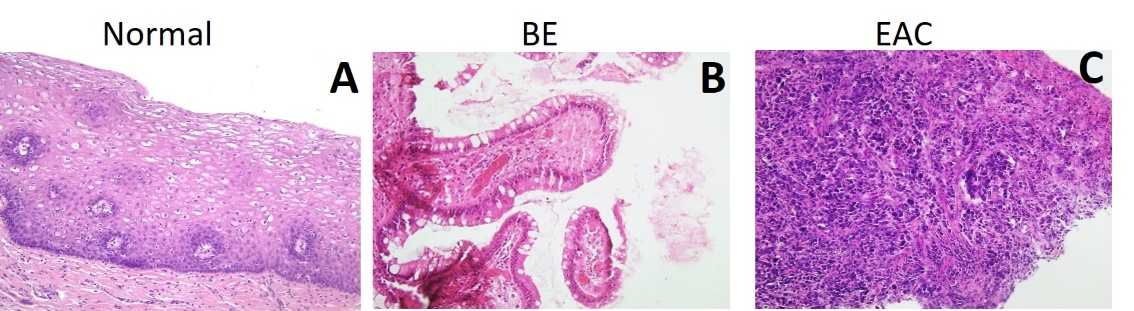


**Supplementary Figure 1**: Hematoxylin and Eosin staining for normal, Barrett’s, and esophageal adenocarcinoma. Esophagus Biopsy of normal esophagus tissue (panel A) showing unremarkable stratified squamous esophageal mucosa. Esophageal biopsy showing columnar cell epithelium with goblet cell/intestinal metaplasia consistent with Barrett’s esophagus without dysplasia (panel B). Esophageal mass biopsy showing poorly differentiated esophageal adenocarcinoma (panel C). All images were taken at 200x magnification. These are the represented images from a total of 23 normal samples, 10 Barrett’s Esophagus samples, and 19 EAC samples.
